# The provision of a trauma bed in theatre recovery and its impact on trauma theatre efficiency: experience from a high-volume trauma unit

**DOI:** 10.1308/rcsann.2023.0106

**Published:** 2024-02-13

**Authors:** J Saleem, O Brown, C Mclean, K Kurzatkowski, S Radha, R Mallina

**Affiliations:** ^1^ Croydon Health Services NHS Trust, UK; ^2^ University of Baghdad Al-Kindy College of Medicine, Iraq

**Keywords:** Quality improvement, Surgery, Trauma, Efficiency, Orthopaedics

## Abstract

**Introduction:**

Inefficiencies in the trauma setting are well known and have been further exacerbated by the COVID-19 pandemic among other factors, resulting in national guidance to aid improvements in resource utilisation. This study introduced a novel surgeon-led intervention, a trauma bed in recovery, with the aim of improving trauma theatre efficiency.

**Methods:**

This quality improvement project was conducted using a Plan Do Study Act (PDSA) methodology and comprised multiple cycles to assess theatre performance. A multidisciplinary team (MDT) approach with relevant stakeholder input enabled intervention implementation, aimed at facilitating ‘golden patient’ arrival in the anaesthetic room as early as possible. The primary outcome was the time at which the first patient entered the anaesthetic room, and the secondary outcome was the number of cases performed each day.

**Results:**

The study period was 1 year and encompassed three PDSA cycles. The intervention achieved its primary outcome by PDSA cycle 1 and its secondary outcome by PDSA cycle 2, demonstrating statistically significantly improved results (*p* < 0.001). A subanalysis assessed the specific impact of the intervention, and demonstrated a significant improvement in both outcomes when the intervention was used as intended (*p* < 0.0005).

**Conclusions:**

A ringfenced trauma bed significantly improved theatre start times and thereby theatre efficiency. This is a simple, pragmatic intervention that benefitted the MDT trauma team while also demonstrating a sustained impact. Given that National Health Service efficiency is at the forefront of national healthcare discourse, we recommend that this intervention is implemented in other trauma units to help provide a solution to this longstanding issue.

## Introduction

Orthopaedic trauma constitutes a large healthcare burden in the United Kingdom, and the National Health Service (NHS) relies on dedicated trauma theatres to manage patients requiring surgery. Considering the time-critical nature of trauma operations, trauma theatre efficiency is vital. Ensuring theatre efficiency is also important given the substantial costs incurred in running trauma theatres.^1^

The existing literature has highlighted inefficiencies in the trauma setting, including late start times and delays in patient changeovers.^2^ The COVID-19 pandemic also disrupted healthcare systems, necessitating the reorganisation of resources to meet demand.^3,4^ This has been evidenced in the trauma theatre setting, with a long-term detrimental effect on theatre efficiency, including a reduction in the number of cases performed daily.^5,6^ These issues have required national guidance to aid resource utilisation improvements across specialties.^7^

Aiming to improve theatre efficiency is not a novel concept, and previous studies have demonstrated that solutions can be found in combatting slow start times and delayed patient turnover.^8^ Furthermore the ‘golden patient’ concept has demonstrated improved start times and operating capacity via the use of preoperative checklists.^9^ Despite this, the pandemic and its effect on trauma services has led to renewed focus on these issues, with recent literature demonstrating the need for innovative processes and increased resource allocation.^10,11^ This experience has been noted at our large district general hospital, with the pandemic increasing the strain on resources and impacting theatre efficiency. There was multidisciplinary team (MDT) agreement on the need for a concerted effort to improve theatre efficiency, particularly once normal service delivery resumed following the lifting of pandemic restrictions. Because numerous previous efforts had failed, a more focused approach on improving the start time of the first case was deemed most effective, with a likely subsequent beneficial impact on the rest of the list. It was hypothesised that implementation of a ringfenced trauma bed in recovery would expedite the start of the first case. Although anecdotal reports have suggested improved theatre efficiency with the use of a trauma bed in recovery, there are no evidenced benefits of its use in the current literature.

This quality improvement project introduced a surgeon-led intervention, a trauma bed in recovery, targeted at facilitating arrival of the ‘golden patient’ in the anaesthetic room as quickly as possible. This novel intervention, in addition to other pre-existing protocols, ultimately aimed to improve trauma theatre efficiency using a Plan Do Study Act (PDSA) service improvement methodology.

## Methods

### Trauma theatre practice pathway before intervention

The trauma service comprises two orthopaedic inpatient wards, one day surgery ward, one trauma theatre that also includes an anaesthetic room, and a shared theatre recovery area. The hospital runs one trauma theatre, operating daily from 8.00am to 5.30pm Monday to Saturday. On Sundays, emergent cases were referred to a shared combined emergency and planned operating department (CEPOD) list.

The preoperative pathway began with identification of the ‘golden patient’; that is, one who is optimised and deemed appropriate to be the first case of the day. Ideally, this was done the night before surgery to ensure that the patient was marked, consented, optimised and relevant investigations were performed. A surgical and anaesthetic review was also performed before the trauma meeting to troubleshoot any patient-related issues.

The trauma meeting started at 8.00am, and the day’s trauma list was discussed in an MDT setting. Thereafter, a team briefing was undertaken in the trauma theatre, attended by the orthopaedic team, anaesthetic team and theatre staff. Troubleshooting of any issues related to the patient, equipment or staffing was also performed. The patient was sent for to arrive in the anaesthetic room to be anaesthetised with any relevant anaesthetic block procedures performed, and the operation commenced. At a suitable time during the operation the next patient was sent for to arrive in the anaesthetic room if appropriate.

Following the operation, the patient recovered in the theatre recovery area, adjacent to the trauma theatre and was sent back to the inpatient wards for postoperative care, or the day surgery unit if the patient was a day case procedure.

### Intervention rationale

The novel intervention in this study was the ringfencing of a trauma bed in theatre recovery aimed at addressing delays and logistical issues that affected start times and patient changeovers. By allowing the surgical team to send for a patient to theatre recovery (adjacent to trauma theatres) before the trauma meeting, several benefits were anticipated. This practice increased the onus on the surgical team to ensure that patients were eligible for early sending to the trauma bed, considering ‘golden patient’ criteria, appropriateness for early surgery and adherence to national guidelines or best practice standards such as the Best Practice Tariff for neck of femur fracture patients. In particular, this focused on patients arriving from inpatient wards or the emergency department who were often situated 15min or more from trauma theatres and required porters for collection; delays sending patients from these areas were commonplace. Moreover, it allowed the anaesthetic team to more quickly review the patient adjacent to theatres. In addition, the intervention aimed to maximise efficiency by enabling the transfer of a second patient on the trauma list to the trauma bed before completion of the previous patient’s surgery, reducing changeover times.

Monitoring of the patient was performed by the theatre recovery nurses before the operation. If any issues were found that precluded the patient being operated on in a timely manner, the patient could be sent back to the inpatient ward for ongoing preoperative management. It should be noted that not every list is eligible for trauma bed use, because some ‘golden patients’ were situated in the day surgery unit adjacent to the theatres and therefore would not need to use the bed.

The intervention was assessed using PDSA methodology, enabling regular assessment of each new interval aimed at improving performance.

### Stakeholder roles

This study required MDT agreement to ensure its successful implementation.

• The orthopaedic team ensured that patients were optimised according to the ‘golden patient’ protocol. They also arranged the early sending of the ‘golden patient’ to the trauma recovery bed, before the trauma meeting, via discussion with the surgery unit reception team at 7.00am.
• The trauma unit reception/portering team agreed to send for/collect the ‘golden patient’ before the trauma meeting, at 7.00am.
• Trauma unit nursing staff agreed to monitor the patient in the trauma recovery bed.
• The anaesthetic team agreed to see the ‘golden patient’ in theatre recovery when applicable.

### PDSA outline

The PDSA outline is given below.

• Initial data collection gauged trauma service performance preintervention, provided a baseline for comparison and outlined existing challenges/areas of improvement.
• PDSA cycle 1 evaluated theatre performance following implementation of the trauma bed specifically for ‘golden patients’.
• PDSA cycle 2 was performed following enhanced use of the trauma bed to include all patients on a trauma list, with the aim of improving transfer times across the list via the same rationale.
• PDSA cycle 3 assessed the intervention’s sustained impact over time.

### Data collection

Data were collected retrospectively to enable a review of preintervention performance. Postintervention performance data were collected prospectively across PDSA cycles to enable comparative performance analysis. To ensure the collection of reliable data, data were collected in a hybrid model, both physically in real time (with members of staff designated for data collection in case of absences) and corroborated with theatre logbooks and clinical software. PDSA collection cycles were not predetermined.

### Patient inclusion/exclusion criteria

Patients of all ages who underwent surgery in our trauma theatre were included in the study. Because the Sunday list is an CEPOD list shared with general surgery, data on this day were excluded. Owing to the unpredictability of trauma, extremes of variations in operative caseloads can occur, and very uncommonly some lists did not achieve full case capacity. To mitigate this variability, if a day did not have a full list the case number was not included in the analysis (this included preintervention lists); however, because the start time of the list would not be affected, these data were still included in the analysis. This aimed to ensure comparisons were made with ‘like for like’ full capacity trauma lists. For example, COVID-19 cancellations had a significant effect on cancellations during PDSA1, leading to numerous trauma lists having reduced case numbers.

**Figure 1:**
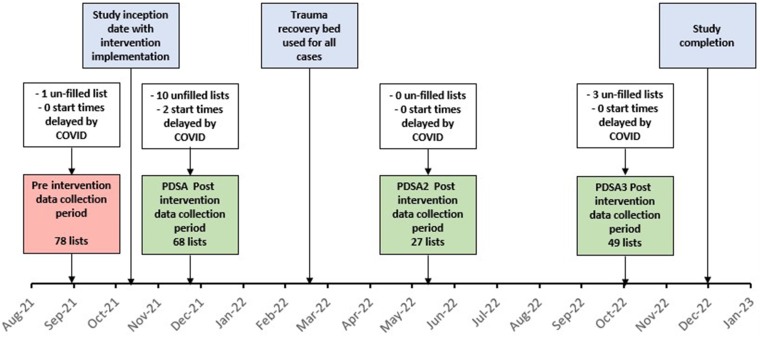
Plan Do Study Act cycle, intervention implementation dates and data collection periods

Figure 1 illustrates the PDSA cycle periods and delineates intervention implementation dates and data collection periods in graphical form.

### Study outcome measures

The primary outcome of this study was to improve the time at which the patient arrived in the anaesthetic room. This was deemed the most suitable primary outcome because the intervention aimed to have a direct effect on facilitating early arrival at the theatre unit. The secondary outcome was to increase the number of cases performed per day. This was deemed a secondary outcome because starting more quickly could increase the ability to perform more cases, but conversely could still be impacted by other variables during the day.

### Data analysis

Outcomes with continuous data were presented as a mean and statistical significance was assessed using parametric t-test. Statistical analysis was carried out using STATA/IC 15.1. Preintervention performance was compared with each PDSA cycle sequentially. Further subanalysis aimed to prove the benefits of using the bed as intended compared with lists where the trauma bed should have been used but was not. This was done by comparing the performance of respective lists.

## Results

The study was conducted between August 2021 and January 2023. Preintervention, 78 trauma lists were analysed. The mean time of arrival of the first patient in the anaesthetic room was 9.15am. The average number of cases performed per trauma list was 3.04. The mean team briefing time was 8.25am. Following implementation of the trauma bed in recovery, the first PDSA cycle, comprising analysis of 68 trauma lists, demonstrated an improved arrival time of 9.01am (*p *< 0.001). The number of cases performed in PDSA cycle 1 increased to 3.29 but did not reach statistical significance. PDSA cycle 2 evaluated the role of the trauma bed being used for all eligible cases in the day’s trauma list. Twenty-seven lists were analysed, and the arrival time of the first patient in the anaesthetic room was further reduced to 8.52am (*p *< 0.0001). The mean case number further increased to 3.67, a statistically significant difference compared with preintervention performance (*p *< 0.0001). During PDSA cycle 3, overall performance reduced slightly, with a mean arrival time of 8.55am and a mean case number of 3.61. However, these improvements remained significantly significant compared with preintervention performance. Table 1 summarises these results.

A subanalysis enabled a more focused assessment of the bed’s impact on performance (Table 2). Across all PDSA cycles, when the bed was used appropriately for the ‘golden patient’ (*n *= 52), the first patient arrived in the anaesthetic room on average 22min earlier (8.48am vs 9.10am; *p *< 0.0001) compared with when the bed should have been used but was not (*n *= 33). Furthermore, when the bed was used appropriately, the number of cases per day was 3.67 compared with 3.07 when the bed was not used (*p *< 0.0005). Across PDSA cycles, the bed was used as intended 60% of the time, with PDSA cycle 2 achieving a 90% successful rate of use across ten eligible lists. Of note, PDSA cycle 2 achieved the best performance across outcomes (Table 3).

**Table 1:** Time of first patient to arrive in the anaesthetic room and number of cases performed per list, when the trauma bed is used appropriately compared with when the trauma bed was not used (when it should have been), across all Plan Do Study Act (PDSA) cycles

	*N*	Mean	*p*-value
Time of first patient to anaesthetic room			
Bed used appropriately	52	8.48am	**<0.00001**
Bed failed to be used	33	9.10am	
Number of cases performed per list			
Bed used appropriately	50	3.67	**<0.0005**
Bed failed to be used	27	3.07	

**Table 2:** Bed use performance delineated by each Plan Do Study Act (PDSA) cycle

Bed use performance	Bed used appropriately	Bed failed to be used	Successful bed use (%)
PDSA 1	20	17	54
PDSA 2	9	1	90
PDSA 3	23	16	59
Total	52	34	60

Assessing PDSA cycle 2, ie use of the trauma bed across the course of an entire list (where appropriate), just under half of all lists used the trauma bed on multiple occasions during a list (12 of 27 in PDSA cycle 2, and 22 of 46 in PDSA cycle 3), demonstrating that the intervention did have scope for use. As noted PDSA cycle 2 and PDSA cycle 3 had the highest number of mean cases per day.

The mean time for the team briefing across the three PDSA cycles was 8.25am.

**Figure 2:**
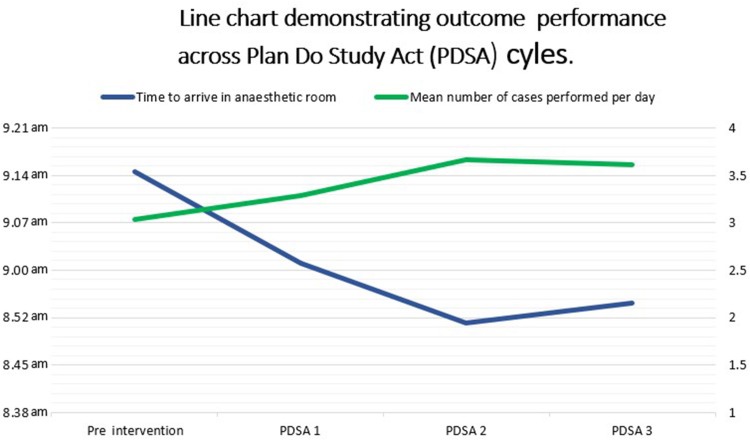
Outcome performance across Plan Do Study Act (PDSA) cycles

Figure 2 shows the key outcomes in graphical form.

**Table 3:** Mean time for patient to enter the anaesthetic room, number of cases performed and theatre efficiency performance, delineated across Plan Do Study Act (PDSA) cycles

Time of first patient to anaesthetic room	*N*	Mean	*p*-value
Preintervention	78	9.15am	
PDSA 1	66	9.01am	**<0.001** (to preintervention)
PDSA 2	27	8.52am	**<0.0001** (to preintervention)
PDSA 3	49	8.55am	**<0.0001** (to preintervention)
Number of cases performed per list			
Preintervention	77	3.04	
PDSA 1	58	3.29	0.0535 (to preintervention)
PDSA 2	27	3.67	**<0.0001** (to preintervention)
PDSA 3	46	3.61	**<0.0001** (to preintervention)

## Discussion

In the context of increasing emphasis on efficiency and resource management within the NHS, this study aimed to evaluate the role of a ringfenced recovery trauma bed in improving theatre efficiency in a busy district general hospital. To our knowledge this is the first study to look at the effect of using a trauma bed in recovery on trauma theatre efficiency. The intervention achieved its primary outcome by PDSA cycle 1, with a statistically significant improvement in the time (more 20min) taken for the patient to reach the anaesthetic bay. The intervention also achieved its secondary outcome, a statistically significant improvement in the number of cases performed per day, which was achieved by PDSA cycle 2. A subanalysis delineated the effect of the bed, demonstrating a significant improvement in both anaesthetic room attendance time and the number of cases per day when the trauma bed was used as intended, compared with when it was not. Furthermore, as bed use increased (PDSA cycle 2, 90%), the results improved compared with when the bed was not more successfully used (PDSA cycle 1, 54%), albeit PDSA cycle 2 had fewer lists. Although the trauma bed could have been used more often, even the evidenced degree of use (60%) highlights its role in improving theatre efficiency.

The success of the intervention can be attributed to several reasons. It provides an onus on the surgical team to ensure that the ‘golden patient’ is optimised as per ‘golden patient’ principles owing to the need to proactively send for the patient before the trauma meeting. Logistically, the bed allows the patient to be brought closer to theatre for porter-free transfer and a more efficient anaesthetic review. This ability was thereafter extrapolated to patients across the trauma list, allowing for patients later in the list to also be sent to the trauma bed. Of note, during this study it was noted that a paediatric ‘golden patient’ often caused significant delays; use of the trauma bed was extended to paediatric cases in PDSA cycles 2 and 3 to improved effect (arrival time improved from 9.12am to 8.56am) while providing a comfortable area for the child and parent to await their procedure. Moreover, because the recovery nursing staff start work at 7.00am, this intervention did not require additional staff support for patient arrival. Throughout the day, the trauma bed did not preclude other patients from using the recovery bays, nor were there cases in which the ‘golden patient’ could not be sent for because of a lack of space. Theoretically if this occurs, the patient would be sent straight to the anaesthetic room as per normal protocol as a failsafe. Of note, only one patient was sent back to the ward during this study because they became unwell. This supports the safety aspect of the intervention. Across multiple MDT and clinical governance meetings, as well as anecdotally, the trauma bed was not highlighted as an issue precluding normal theatre recovery logistics. Although use of the bed relies on MDT agreement, it is an otherwise pragmatic, cost-free intervention that is surgeon led and could be implemented in any trauma unit nationally. As demonstrated in the literature, improving efficiency across an entire trauma list is difficult because of the multifactorial nature of the trauma service; however, a strength of our intervention is its focus on improving theatre start times and its resultant effect on the rest of the list. Addressing inefficiencies across every facet of a trauma list was not the aim of this study and would be very difficult to solve without wholesale evaluation of all aspects of the list.

The strengths of this study include its innovative approach to addressing theatre inefficiency; to the authors’ knowledge this has not been studied before, nor is it a well-explored topic despite its relevance to healthcare systems. The PDSA model of improvement is an established framework for implementing change, and this project was able to assess three cycles of interventional change.^12^ Analysis of multiple PDSA cycles allowed us to measure the effects of the intervention at each stage as well as its long-term effect over more than a year. The differences in the number of lists analysed in the PDSA cycles are of note. Preintervention, 78 lists, a large number, were analysed to give a detailed description of practice before implementation of the trauma bed. Quality improvement relies on careful data collection, therefore data collection periods occurred when a staff member who was fully aware of the study was present to ensure quality data collection accuracy. These data collection periods were still relevant to ensuring assessment of the intervention.

### Study limitations

There are limitations to this study. We acknowledge the role of confounders when improving service provision and quality improvement. To our knowledge, no other significant intervention was implemented and no significant change to the trauma service occurred during the study period, particularly one that could have affected start time outcomes. Confounding in the form of weekly variations in trauma workload and COVID disruption was mitigated for as discussed in the Methods. Furthermore, this study acknowledges the potential for observation bias in the reporting of data, because reporters were not blinded to data collection periods. As noted, the trauma bed was used as intended in 60% of lists. Although 100% usage would have been ideal, trauma is unpredictable and last-minute switches to the ‘golden patient’, unforeseen MDT issues and other logistical issues meant that 100% usage was and is likely to be unattainable. Despite this, the subanalysis demonstrated favourable performance in lists that used the bed compared with those that did not.

Overall, this study demonstrates that a ringfenced trauma bed in recovery improves theatre start times and efficiency, enabling a greater number of cases to be undertaken. Being a surgeon-led concept places enhanced responsibility and onus on the surgical team to ensure lists run smoothly, but also supports MDT members such as the anaesthetic team in carrying out their roles. It is a simple, pragmatic, cost-free intervention that has proven to be safe and not caused any known disruption to our operating pathway, while also demonstrating a sustained impact across multiple PDSA cycles throughout a 1-year period. As resource allocation constraints and the burden of trauma, particularly among the elderly population, continue to rise, as well as the prolonged recovery following the COVID pandemic, NHS efficiency is at the forefront of national healthcare discourse. This study recommends that this intervention, in tandem with other novel ideas, are implemented across other trauma units to improve the trauma service provided to patients and staff, and help reduce widespread inefficiencies.

## References

[1] NHS Institute for Innovation and Improvement. The Productive Operating Theatre. www.institute.nhs.uk/quality_and_value/productivity_series/the_productive_operating_theatre.html (cited June 2015).

[2] Rymaruk S, Buch K. How is time used within the orthopaedic trauma theatre? *J Perioper Pract* 2015; **25**: 188–191. 26717586 10.1177/175045891502501001

[3] Halawi MJ, Wang DD, Hunt TR. What’s important: weathering the COVID-19 crisis. *J Bone Joint Surg* 2020; **102**: 759–760. 32379115 10.2106/JBJS.20.00419 PMC7219834

[4] Cao Y, Li Q, Chen J *et al.* Hospital emergency management plan during the COVID-19 epidemic. *Acad Emerg Med* 2020; **27**: 309–311. 32124506 10.1111/acem.13951 PMC7159322

[5] Khadabadi NA, Logan PC, Handford C *et al.* Impact of COVID-19 pandemic on trauma theatre efficiency. *Cureus* 2020; **12**: e11637. 33376649 10.7759/cureus.11637 PMC7755676

[6] Arshad F, Hanif UK, Arshad A *et al.* Orthopaedic trauma theatre efficiency in the COVID-19 pandemic: are we returning to normality? *Cureus* 2021; **13**: e13221. 33728171 10.7759/cureus.13221 PMC7946018

[7] COVID-19: Good Practice for Surgeons and Surgical Teams. Professional and Clinical Standards. Originally published March 2020.

[8] Rodriguez T, Wolf-Mandroux A, Soret J *et al.* Compared efficiency of trauma versus scheduled orthopaedic surgery operating rooms in a university hospital. *Orthop Traumatol Surg Res* 2019; **105**: 179–183. 30639174 10.1016/j.otsr.2018.10.019

[9] Key T, Reid G, Vannet N *et al.* ‘Golden patient’: a quality improvement project aiming to improve trauma theatre efficiency in the Royal Gwent Hospital. *BMJ Open Qual* 2019; **8**: e000515. 10.1136/bmjoq-2018-000515 PMC6440604 30997419

[10] Luengo-Alonso G, Pérez-Tabernero FG, Tovar-Bazaga M *et al.* Critical adjustments in a department of orthopaedics through the COVID-19 pandemic. *Int Orthop* 2020; **44**: 1557–1564. 32474718 10.1007/s00264-020-04647-1 PMC7260474

[11] Virani S, Faria G, Housden P. Efficiency changes in orthopaedic trauma surgery and implications for resource allocation. *Br J Hosp Med (Lond)* 2021; **82**: 1–6. 10.12968/hmed.2021.0060 34431355

[12] Quality, service improvement and redesign tools: Plan, Do, Study, Act (PDSA) cycles and the model for improvement. https://www.england.nhs.uk/wp-content/uploads/2022/01/qsir-pdsa-cycles-model-for-improvement.pdf

